# FACT, the Bur Kinase Pathway, and the Histone Co-Repressor HirC Have Overlapping Nucleosome-Related Roles in Yeast Transcription Elongation

**DOI:** 10.1371/journal.pone.0025644

**Published:** 2011-10-12

**Authors:** Jennifer R. Stevens, Allyson F. O'Donnell, Troy E. Perry, Jeremy J. R. Benjamin, Christine A. Barnes, Gerald C. Johnston, Richard A. Singer

**Affiliations:** 1 Department of Biochemistry and Molecular Biology, Dalhousie University, Halifax, Nova Scotia, Canada; 2 Department of Microbiology and Immunology, Dalhousie University, Halifax, Nova Scotia, Canada; Texas A&M University, United States of America

## Abstract

Gene transcription is constrained by the nucleosomal nature of chromosomal DNA. This nucleosomal barrier is modulated by FACT, a conserved histone-binding heterodimer. FACT mediates transcription-linked nucleosome disassembly and also nucleosome reassembly in the wake of the RNA polymerase II transcription complex, and in this way maintains the repression of ‘cryptic’ promoters found within some genes. Here we focus on a novel mutant version of the yeast FACT subunit Spt16 that supplies essential Spt16 activities but impairs transcription-linked nucleosome reassembly in dominant fashion. This Spt16 mutant protein also has genetic effects that are recessive, which we used to show that certain Spt16 activities collaborate with histone acetylation and the activities of a Bur-kinase/Spt4–Spt5/Paf1C pathway that facilitate transcription elongation. These collaborating activities were opposed by the actions of Rpd3S, a histone deacetylase that restores a repressive chromatin environment in a transcription-linked manner. Spt16 activity paralleling that of HirC, a co-repressor of histone gene expression, was also found to be opposed by Rpd3S. Our findings suggest that Spt16, the Bur/Spt4–Spt5/Paf1C pathway, and normal histone abundance and/or stoichiometry, in mutually cooperative fashion, facilitate nucleosome disassembly during transcription elongation. The recessive nature of these effects of the mutant Spt16 protein on transcription-linked nucleosome disassembly, contrasted to its dominant negative effect on transcription-linked nucleosome reassembly, indicate that mutant FACT harbouring the mutant Spt16 protein competes poorly with normal FACT at the stage of transcription-linked nucleosome disassembly, but effectively with normal FACT for transcription-linked nucleosome reassembly. This functional difference is consistent with the idea that FACT association with the transcription elongation complex depends on nucleosome disassembly, and that the same FACT molecule that associates with an elongation complex through nucleosome disassembly is retained for reassembly of the same nucleosome.

## Introduction

The packaging of DNA into chromatin, mainly in the context of nucleosomes, restricts access to the DNA template. Several proteins or protein complexes regulate access to DNA for transcription [1], [2]. Among these is a nuclear heterodimer named FACT(facilitates chromatin transcription), a histone chaperone whose subunits are encoded throughout the eukaryotic lineage. While FACT has recently been implicated in the transcription of rRNA genes by RNA polymerase I and small-RNA genes by RNA polymerase III [3], more is known about the involvement of FACT in facilitating transcription of protein-coding genes by RNAPII and its accessory proteins [4].

FACT has several activities during transcription. Most notably, FACT is involved in diminishing the nucleosomal barrier to transcription that is encountered by RNAPII, and in this way facilitates transcription elongation. This outcome is achieved in part by the destabilization of normal nucleosomal structure, which can involve the reorganization of normal histone protein interactions and/or the displacement of histones [2]. FACT can destabilize a nucleosome during *in vitro* transcription by dissociating one histone H2A–H2B heterodimer from the rest of the nucleosome [5], [6]. FACT can also reorganize nucleosomal structure without H2A–H2B displacement [7]. FACT and/or its subunits bind intact nucleosomes, the H2A–H2B dimer, and the histone (H3–H4)_2_ tetramer [5], [8]–[10], and genetic studies of yeast FACT indicate a role for FACT in nucleosome disassembly or reorganization through modulation of H2A–H2B:(H3–H4)_2_ interactions [11], [12]. Furthermore, the Spt16 subunit of FACT can be mutated to relieve the transcriptional effects of a histone H3 mutation, further evidence for FACT–nucleosome interactions [13], [14]. FACT co-purifies with transcription complexes and localizes to transcribed regions, suggesting that FACT travels with RNAPII to facilitate access to nucleosomal DNA during transcription elongation [15]–[18].

FACT also participates in the nucleosome reassembly that takes place in the wake of the transcription complex, as evidenced by the transcription-dependent loss of histones from transcribed regions in yeast cells mutant for FACT subunit Spt16 [19], [20]. Another readout of transcription-linked nucleosome reassembly is the maintenance of chromatin repression of transcription from ‘cryptic’ promoter sequences that exist within some yeast genes [21]. Sequences internal to many transcribed regions have the potential to be sites of transcription initiation, but are repressed by normal nucleosome structure. The maintenance of this repression during transcription depends on the efficient reestablishment of nucleosome structure after the passage of RNAPII. FACT mutations can allow transcription initiation at cryptic promoter sites [16], [21]–[24]; indeed, a genome-wide survey found that over 15% of yeast genes harbor cryptic promoters whose repression depends on FACT [25]. FACT activity, through its ability to mediate the transcription-linked restoration of nucleosome structure, is thus necessary for the fidelity of transcription initiation.

FACT has effects on transcription initiation that are more direct. The yeast FACT subunit Spt16 physically interacts with the general transcription factor TFIIE, and shows genetic interactions with the general transcription initiation factor TFIIA and with TATA-binding protein TBP, which binds TFIIA and promoter DNA for transcription initiation [26], [27]. Mutating the Spt16 subunit of FACT can decrease TBP binding to promoters *in vivo*, while *in vitro* assays show that FACT facilitates the cooperative binding of TBP and TFIIA to promoter sequences that are in a nucleosomal configuration [16], [27], [28]. This effect of FACT on TBP binding reflects several activities, including chromatin disassembly at promoters [29]–[31], which may in some cases reflect the nucleosome dynamics accompanying regulatory transcription across a promoter [32]. FACT subunits also have physical and functional interactions with gene-specific transcription activators [29], [31], [33]–[35].

Significant advances in understanding Spt16 functions, and thus most likely FACT functions, have been made through the study of *spt16* mutations that were identified because they cause temperature sensitivity for yeast cell proliferation [8]. Such mutational consequences need not be specific to transcription: FACT and/or its subunits are also implicated in DNA replication and heterochromatin function [36]–[39]. Moreover, many temperature-sensitive *spt16* mutations destabilize the mutant Spt16 protein, making it difficult to distinguish inadequate but normal Spt16 function from mutationally impaired function [40], [41]. To avoid potentially confounding effects due to protein stability, we decided to study the functional relationships of Spt16 by focusing on mutant alleles of the yeast *SPT16* gene that affect function in a dominant manner; our reasoning was that a mutant Spt16 protein with a dominant effect would be expected to be relatively stable [42]. The *spt16-E857K* and *spt16-E763G* substitution mutations were identified through the impaired control over inappropriate transcription initiation that they cause as a consequence of defective nucleosome reassembly in the wake of transcribing RNAPII: each of these mutations, in dominant (or co-dominant) fashion, activates functional transcription from cryptic promoters within certain reporter genes [42]. Nonetheless, the Spt16-E857K and Spt16-E763G mutant proteins also supply all essential activities of Spt16 [42]. These observations, coupled with the knowledge that the FACT heterodimer contains a single Spt16 subunit [43], [44], indicates that each mutant version of FACT retains important interactions, but is deficient in interactions that facilitate efficient nucleosome reassembly.

The *spt16-E857K* mutant allele was used here in a genetic study to explore functional interactions with several components that influence transcription elongation. These experiments indicated that Spt16, and therefore FACT, has activities that parallel those of other conserved factors that facilitate transcription elongation, including the elongation factors Bur1–Bur2, Spt4–Spt5 and Paf1C, and the HirC corepressor of histone gene expression. Furthermore, several deleterious genetic interactions involving *spt16-E857K* were alleviated by preventing the Rpd3S-mediated deacetylation of histones that normally occurs during transcription elongation. These findings suggest not only that Spt16 carries out chromatin-related activities during transcription elongation, but also that these activities overlap functionally those of a Bur kinase/Spt4–Spt5/Paf1C pathway.

## Results

### Novel dominant Spt16 mutants relieve repression of ‘cryptic’ promoters

The transcription process involves the disassembly of nucleosomes for access to DNA, followed by the reassembly of nucleosomes in the wake of the transcription-elongation complex. Impaired transcription-linked nucleosome reassembly can allow aberrant transcription initiation from sequences (‘cryptic promoters’) within the bodies of certain genes. In an earlier study we described the identification and partial characterization of novel mutant alleles of the *SPT16* gene that, in dominant fashion, allow effective transcription from cryptic promoters in certain reporter genes [42]. We report here that this dominant phenotype is also caused by several more *spt16* mutant alleles (Table 1, which also lists the mutant alleles described previously).

**Table 1:** *spt16* Mutant alleles that activate cryptic promoters in dominant fashion (1).

		In *SPT16* cells (2)		As the sole *spt16* allele (2)			
Allele	Substitution(s)	High-temp. growth (37°C)	Growth on 100 mM HU (30°C)	Essential function (30°C)	Cryptic-promoter activity (30°C)	High-temp. growth (37°C)	Growth on 100 mM HU (30°C)
*SPT16*	—	+	+	+	−	+	+
*spt16-E857K*	E857K	+	nd (4)	+	+	+	+/−
*spt16-E763G*	E763G	+	nd	+	+	+	+
*spt16-312*	E763G, R784G, S819P	+	+	+	+	−	+
*spt16-319*	L804P, L946S, E1004G	+	+	+	+	−	−
*spt16-L804P*	L804P	+	+	+	+	−	−
*spt16-216* (3)	T677M, R686G, K692E	+	+	−	na (5)	na	na
*spt16-Q682R,D770G*	Q682R, D770G	+	nd	+	+	+	+
*spt16-Q682R,T772P*	Q682R, T772P	+	nd	+	+	+	nd
*spt16-D766G*	D766G	+	nd	+	+	+	−
*spt16-E794V*	E794V	+	+	−	na	na	na
*spt16-D884G*	D884G	nd	+	+	+	+	−
*spt16-V902F,N927D*	V902F, N927D	+	+	−	na	na	na
*spt16-F944V,F945S* (3)	F944V, F945S	+	+	−	na	na	na

^**(1)**^All mutant alleles listed here, on low-copy plasmids, activate the reporter genes *his4-912δ*, *lys2-128δ*, and *GAL1pr-FLO8-HIS3* at 30°C in *SPT16* cells (strains AW11-9a and  FY2393). Some data for *spt16-E857K*, *spt16-E763G*, *spt16-312*, and *spt16-319* are from [42].

^**(2)**^ *SPT16* cells: strains AW11-9a and FY2393; *spt16Δ* cells: strain KanB-[plasmid].

^**(3)**^Not dissected further.

^**(4)**^Not determined.

^**(5)**^Not applicable.

A useful reporter of chromatin status is the *prGAL1-FLO8-HIS3* gene, which was constructed to have functional transcription of its *HIS3* ORF possible only through transcription initiation at a cryptic internal promoter, within *FLO8* ORF sequences, that is normally repressed by chromatin (nucleosome) structure [25]. The controllable *GAL1* promoter driving this *FLO8-HIS3* reporter gene allows evaluation of the requirement for transcription across the cryptic internal promoter from the upstream *GAL1* promoter, as expected for a situation of promoter activation through faulty nucleosome reassembly in the wake of RNAPII passage. All plasmid-borne *spt16* mutant alleles listed in Table 1 activated the *prGAL1-FLO8-HIS3* reporter gene in dominant fashion, in a manner dependent on *GAL1* promoter activity. Similarly, in cells lacking the chromosomal *SPT16* gene the novel *spt16* mutant alleles that supply essential function activated this reporter gene in a manner dependent on *GAL1* promoter activity (Table 1). Thus all of the novel *spt16* mutant alleles have effects consistent with inefficient transcription-linked nucleosome reassembly.

As indicated in Table 1, we found that several mutations selected by transcription-linked nucleosome-reassembly effects also display other known phenotypes of *spt16* mutations [8]. Thus other biological activities of Spt16 may also be compromised by some of these mutations, including *spt16-E857K*.

### A screen for deleterious genetic interactions with *spt16-E857K* identified *bur2*

We used the *spt16-E857K* mutant allele that interferes with transcription-linked nucleosome reassembly in a screen for deleterious genetic interactions, indicative of a common downstream function for the proteins affected by the two mutations [45]. Mutations that are deleterious in combination with *spt16-E857K* can show how Spt16 activity relates to the activities of other factors.

**Figure 1:**
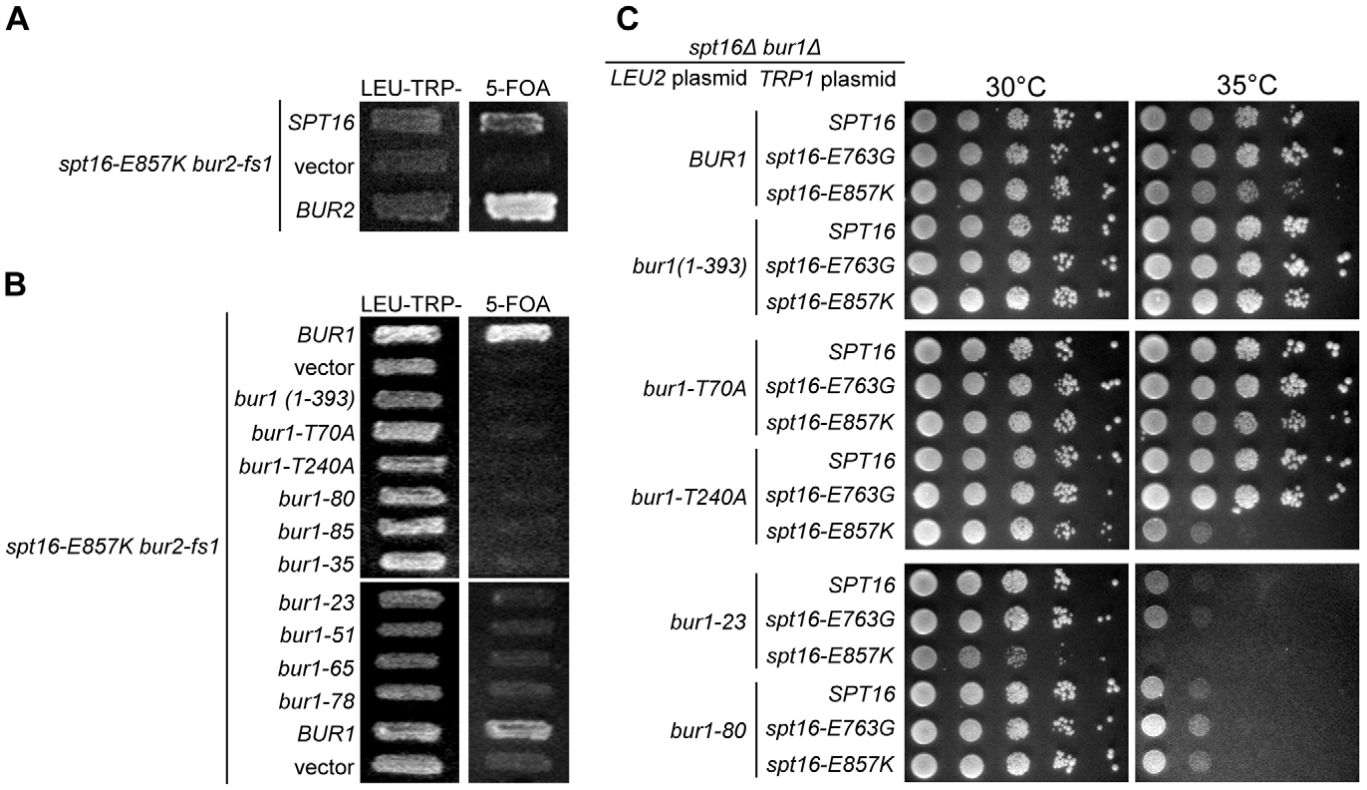
Cells relying of Spt16-E857K need significant Bur kinase activity. (A) The inviability of *spt16-E857K bur2-fs*1 double-mutant cells is complemented by the *SPT16* or *BUR2* gene. Using a plasmid-shuffling approach, *spt16Δ bur2-fs1* cells (strain AFO416), deleted for the chromosomal *SPT16* ORF but kept alive by *spt16-E857K TRP1* and *SPT16 URA3* plasmids, were transformed with a *LEU2* plasmid carrying the indicated gene, and derivatives that failed to inherit the *SPT16 URA3* plasmid were selected on 5-FOA medium. Only cells harbouring a *LEU2*-based plasmid that alleviates the *spt16-E857K bur2-fs1* synthetic lethality are able to grow on 5-FOA medium. (B) Increased Bur1 activity suppresses the inviability of *spt16-E857K bur2-fs*1 double-mutant cells. Cells of strain AFO416 were transformed with *BUR1 LEU2* or *bur1 LEU2* plasmids before 5-FOA selection for cells that had failed to inherit the *SPT16 URA3* plasmid. (C) *spt16-E857K* mutant cells need significant Bur1 activity for growth. Plasmid-shuffle procedures were used to create derivatives of *spt16Δ bur1Δ* double-mutant cells (strain bur1Δ68Δ3C) that carry *BUR1* (or *bur1*) *LEU2* plasmids, and *SPT16* (or *spt16*) *TRP1* plasmids. These derivatives (strains JS312 through JS329) were then grown to stationary phase in liquid culture and normalized for cell concentration, and 10-fold serial dilutions were spotted on selective solid medium for growth at the indicated temperatures.

Through a plasmid-loss procedure and two-color discrimination system for screening yeast colonies [46], [47] we identified five derivatives that failed to grow when relying on the Spt16-E857K mutant protein, but grew well if the cells also contained normal Spt16. These derivatives therefore harbor mutations that have deleterious interactions with *spt16-E857K* effects that are recessive. For one derivative from the screen, lethality was alleviated by genomic plasmids and subclones containing the *BUR2* gene, suggesting the presence of a *bur2* mutation (Figure 1A). Indeed, this *BUR2* locus (*bur2-fs1*) contains an extra G residue (coding strand) following ORF position 253, causing an R85K substitution and a frameshift that brings into register a stop codon, resulting in a truncated protein. This Bur2 fragment is unlikely to be functional: *SPT16 bur2-fs1* mutant cells have a slow-growth phenotype analogous to that of *bur2Δ* cells (Figure 1A). Thus Spt16 and Bur2 have a common downstream function.

### *spt16-E857K* interacts genetically with both subunits of Bur kinase

Bur2 is involved in transcription elongation as the regulatory subunit of the Bur1–Bur2 cyclin-dependent kinase; Bur1 is the catalytic subunit. Deletion of *BUR2* decreases Bur1 activity; conversely, many *bur2Δ* effects are alleviated by increased *BUR1* expression [48]. We found that the deleterious genetic interaction between *bur2* and *spt16-E857K* (in cells with a chromosomal *BUR1* gene) was also relieved by increased Bur1 expression from a low-copy *BUR1* plasmid, although not by several partially functional Bur1 mutants [49] expressed in the same way (Figure 1B). This finding suggests that the lethality of *spt16-E857K bur2* double-mutant cells is due to inadequate Bur kinase activity.

Using cells with normal Bur2 function, we tested several *bur1* mutations [49] for genetic interactions with *spt16-E857K*, using a plasmid-shuffling approach. In combination with *spt16-E857K* none of the *bur1* mutations was lethal, but growth was impaired by two mutations with decreased Bur kinase activity: the temperature-sensitive mutant allele *bur1-23* and *bur1-T240A*, mutated to eliminate an activating phosphorylation site (Figure 1C). These observations, along with our *bur2* findings, suggest that the *spt16-E857K* mutation compromises a specific Spt16 activity, so that phosphorylation by Bur kinase becomes essential; that is, Spt16 and phosphorylation by Bur kinase have a common downstream effect. Based on these findings we decided to take an *in vivo* genetic approach to understand the functional relationship between Spt16 and the transcription elongation factor Bur kinase.

### No genetic interaction between *spt16-E857K* and CTDK-1, an RNAPII CTD kinase

One Bur kinase substrate is RNAPII. Bur kinase phosphorylates the largest subunit of RNAPII on serine-2 of its C-terminal-domain (CTD) repeat to stimulate transcription elongation [50], [51]. In this activity, Bur kinase may be a yeast ortholog of mammalian P-TEFb. Yeast cells have another putative P-TEFb ortholog, CTDK-1, which also phosphorylates Ser-2 of the CTD repeat [52]. We found that eliminating the CTDK-1 catalytic subunit by a *ctk1Δ* gene deletion failed to affect the growth of *spt16-E857K* cells (data not shown). This observation is consistent with other work showing that CTDK-1 and Bur kinase have distinct functions: they are recruited to the transcription complex through distinct sequential mechanisms, and phosphorylate distinct spectra of substrates in addition to the RNAPII CTD [50], [52], [53]. The genetic interaction between *spt16-E857K* and Bur kinase may therefore implicate a phosphorylation substrate other than the RNAPII CTD.

### *spt16-E857K* does not impair the Rad6–COMPASS pathway

**Figure 2:**
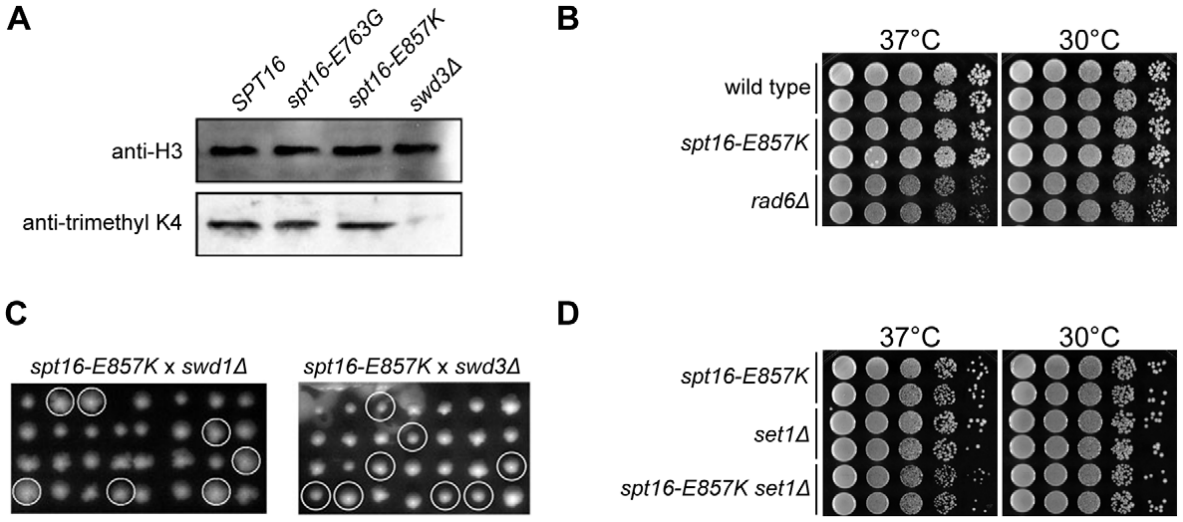
Rad6–COMPASS impairment is not responsible for the Bur-kinase genetic interaction with *spt16-E857K*. (A) Neither *spt16-E857K* nor *spt16-E763G* affect H3K4 trimethylation. Whole cell extracts were prepared from Y2454-WT68, -E857K, and -E763G cells, and equal amounts of extract, as determined by Bradford assays, were resolved electrophoretically and immunoblotted with antibodies against total H3 (top) and H3 trimethylated at K4 (bottom). (B) No genetic interactions between *spt16-E857K* and *rad6Δ*. Haploid genetic segregants from diploid cells made heterozygous for *spt16-E857K* and *rad6Δ* by *RAD6* gene replacement were sporulated and tetrad analysis was carried out. Representative segregants were grown to stationary phase in liquid culture and normalized for cell concentration, and 10-fold serial dilutions were spotted on rich medium for further growth at the indicated temperatures. (C) No genetic interactions between *spt16-E857K* and the Swd1 or Swd3 subunits of COMPASS. Diploid cells (Y2454-E857K×BY4741 *orfΔ*) heterozygous for *spt16-E857K* and *swd1Δ* or *swd3Δ* were sporulated, and the spores from complete tetrads (arranged vertically) were germinated and grown on rich solid medium. Double-mutant segregants containing *spt16-E857K* and either *swd1Δ* or *swd3Δ* are circled. (D) No genetic interactions between *spt16-E857K* and the Set1 catalytic subunit of COMPASS. Diploid cells (Y2454-E857K×BY4741 *set1Δ*) heterozygous for *spt16-E857K* and *set1Δ* were sporulated and tetrad analysis was carried out. Representative segregants were grown to stationary phase and normalized for cell concentration, and 10-fold serial dilutions were spotted on rich solid medium for further growth at the indicated temperatures.

Another target of Bur kinase is Rad6. Phosphorylation of Rad6 promotes histone H2B ubiquitination and the consequent recruitment of the histone methyltransferase COMPASS and trimethylation of histone H3 on lysine 4 (K4), a hallmark of transcribed regions [54], [55]. Our western blots showed no difference in H3K4 trimethylation levels between cells containing normal or mutant Spt16, indicating that the *spt16-E857K* mutation does not impair Rad6 or COMPASS function (Figure 2A).

We investigated the possibility that the *spt16-E857K* mutation might impair an Spt16 activity that overlaps that of H2B ubiquitination or H3K4 methylation by analyzing double-mutant derivatives created by standard genetic procedures. The *rad6Δ* mutation itself causes both cold sensitivity and temperature sensitivity [56] and all of our *rad6Δ* single-mutant segregants exhibited sensitivity to high and low growth temperatures, to the same degree as the double-mutant derivatives (Figure 2B). There was no significant genetic interaction between *spt16-E857K* and *rad6Δ*.

The lack of genetic interactions in *spt16-E857K rad6Δ* cells was mirrored by the lack of genetic interactions between *spt16-E857K* and the *set1Δ*, *swd1Δ*, and *swd3Δ* deletions eliminating components of COMPASS (Figure 2C, D). The *set1Δ* deletion eliminates the methyltransferase subunit, and cells lacking Swd1 or Swd3 do not methylate H3K4 [57]. This finding that *spt16-E857K* failed to impair the growth of COMPASS mutants contrasts with the strong genetic interaction between *set1Δ* and *spt16-11*, a mutant allele encoding a temperature-sensitive Spt16 mutant protein [28]. Evidently the Spt16-E857K mutant protein retains the Spt16 function that overlaps that of COMPASS, and is thus selectively impaired for function. The genetic interactions between *spt16-E857K* and Bur-kinase mutations are due to a consequence of Bur kinase activity other than Rad6-mediated H2B ubiquitination or H3K4 methylation.

### *spt16-E857K* impairs the growth of cells mutant for the Spt4–Spt5 complex

**Figure 3:**
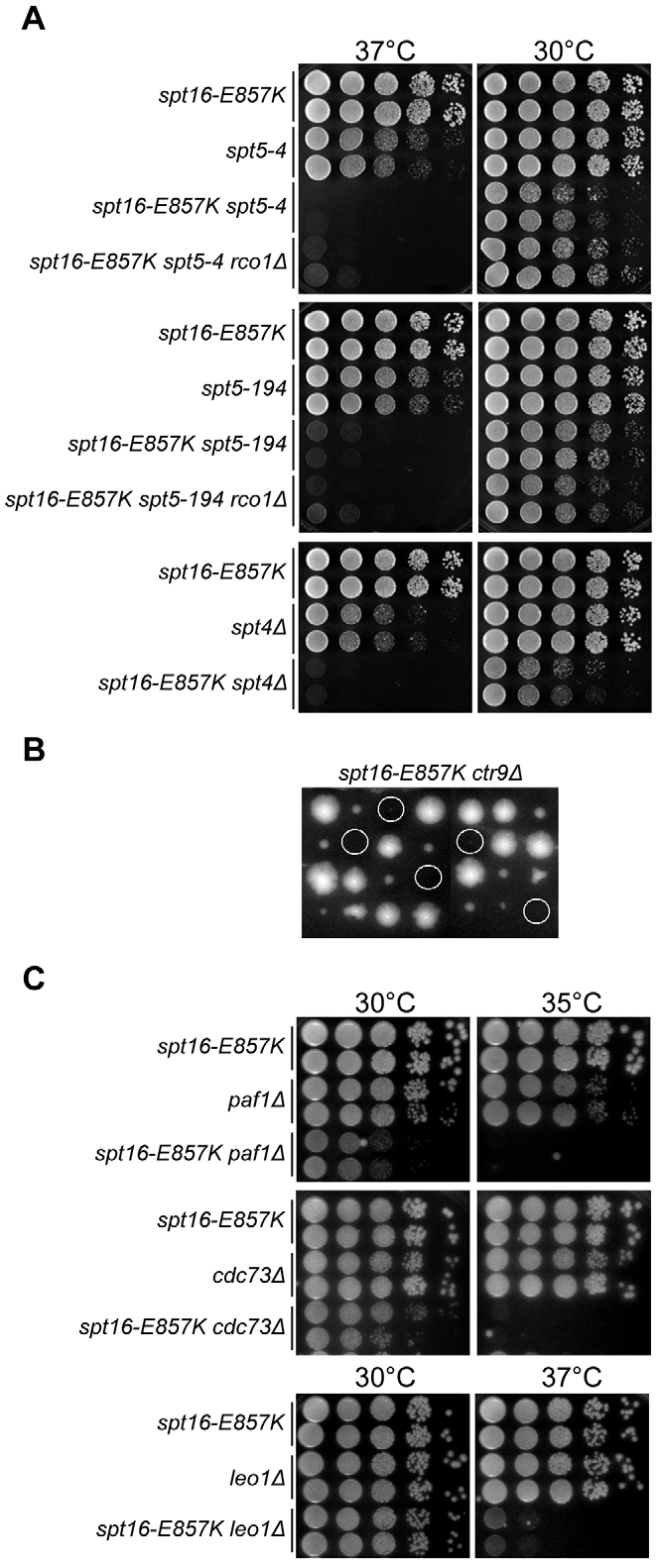
The *spt16-E857* mutation increases reliance on Spt4–Spt5 and Paf1C. (A) Deleterious genetic interactions between *spt16-E857K* and mutations affecting Spt4–Spt5. Cells of strain JS338 were crossed with those of strains JS334, FY362, and FY1668, the resulting diploids were sporulated, and tetrad analysis was carried out. Representative segregants were grown to stationary phase and normalized for cell concentration, and 10-fold serial dilutions were spotted on rich solid medium for further growth at the indicated temperatures. (B) Lethal genetic interaction with the Paf1C component Ctr9. Y2454-E857K×KY693 diploid cells heterozygous for *spt16-E857K* and *ctr9Δ* were sporulated, and the spores from complete tetrads (arranged vertically) were germinated and grown on rich solid medium. Positions of the *spt16-E857K ctr9Δ* double-mutant segregants are circled. (C) Deleterious genetic interactions with Paf1C components. Representative segregants of each indicated genotype were grown to stationary phase and normalized for cell concentration, and 10-fold serial dilutions were spotted on rich solid medium for further growth at the indicated temperatures.

Another target of Bur kinase is the Spt4–Spt5 complex (termed DSIF [DRB-sensitivity inducing factor] in metazoans), which is involved in transcription elongation and counteracts the tendency for nucleosomes to cause transcription arrest [58]. The Spt5 protein contains tandem repeats in its C terminus that are phosphorylated by Bur kinase [51], [59]. We found that the *spt5-4* and *spt5-194* mutant alleles [60], [61] impair growth in combination with *spt16-E857K*, suggesting a common function (Figure 3A). We also found genetic interactions between *spt16-E857K* and an *spt4Δ* deletion eliminating the binding partner of Spt5 (Figure 3A). Thus Spt16 has a function in common with Spt4–Spt5, and this function is impaired by the *spt16-E857K* mutation.

### Genetic interactions between *spt16-E857K* and mutations affecting Paf1C

A downstream effector of Spt4–Spt5 is the multi-subunit complex Paf1C, which is involved in the elongation stages of transcription, associates with FACT, Spt4–Spt5, and RNAPII, and localizes throughout the coding regions of genes. Bur kinase, Spt5 phosphorylation, and the Spt4 protein are important for this Paf1C localization and for Paf1C association with RNAPII, suggesting that Spt4–Spt5 is a platform for Paf1C recruitment [62]. Paf1C facilitates nucleosome disassembly upon the induction of transcription [63].

We saw impaired growth for *spt16-E857K* cells lacking Paf1C components (Figure 3B). The most severe impairments were seen for *spt16-E857K ctr9Δ* double-mutant cells, which failed to grow (Figure 3B), and *spt16-E857K paf1Δ* double-mutant cells, which grew poorly even at 30°C (Figure 3B). Moderate growth impairments were seen for both *spt16-E857K cdc73Δ* and *spt16-E857K leo1Δ* double-mutant cells (Figure 3B). An analogous spectrum of effects has been reported for genetic interactions between Paf1C gene deletions and *spt16-197*, a point mutation that destabilizes the mutant Spt16 protein in temperature-dependent fashion [24], [64]. In general, Paf1C has complex genetic interactions, and the phenotypes of *ctr9Δ* and *paf1Δ* cells are more severe than those of *cdc73Δ* or *leo1Δ* cells [65].

### Set2 and Rpd3S intensify the *spt16-E857K bur2Δ* genetic interaction

The growth of *bur1Δ* cells is markedly improved by several gene deletions (*set2Δ*, *rpd3Δ*, *sin3Δ*, *eaf3Δ* and *rco1Δ*) eliminating members of a histone-modification pathway distinct from the COMPASS pathway described above [66], [67]. In this pathway, the Set2 protein is recruited to transcriptionally engaged RNAP II phosphorylated on serine 2 of its CTD repeats; Set2 then methylates lysine 36 of histone H3 (H3K36) on nucleosomes that are reassembled after RNAPII transit. H3K36 dimethylation allows nucleosome binding by the Rpd3S complex (comprising Rpd3, Sin3, Eaf3, and Rco1), which deacetylates nucleosomal histones to restore a transcriptionally repressive chromatin environment [68].

The observations that Rpd3S deacetylation activity presents a significant problem for cells with impaired Bur kinase suggest that Bur kinase fosters transcription in part by counteracting the effects of a repressive chromatin environment. The *spt16-E857K bur2Δ* synthetic lethality reported here could therefore reflect different but complementary roles played by FACT and Bur kinase in overcoming the repressive effects of deacetylated histones resulting from Rpd3S activity. This hypothesis was tested genetically.

**Figure 4:**
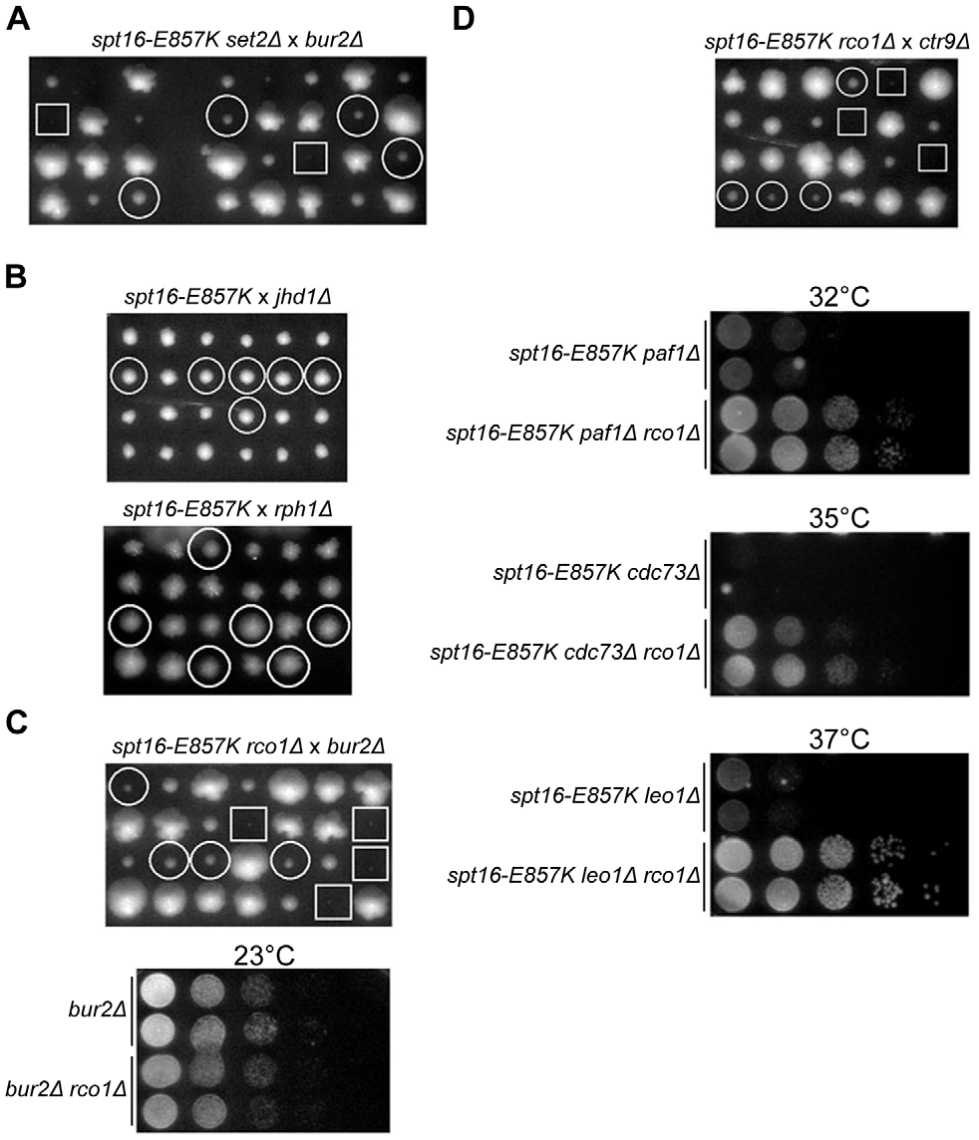
Rpd3S function intensifies deleterious *spt16-E857K* genetic interactions. (A) H3K36 methylation by Set2 is detrimental in *spt16-E857K bur2Δ* cells. Diploid cells heterozygous for *spt16-E857K*, *bur2Δ*, and *set2Δ* were sporulated, the spores from complete tetrads (arranged vertically) were germinated and grown on rich solid medium. *spt16-E857K bur2Δ set2Δ* triple-mutant segregants are circled, while the positions of *spt16-E857K bur2Δ* double-mutant segregants are marked with squares. (B) No obvious benefit of H3K36 demethylation by Jhd1 or Rph1 in *spt16-E857K* cells. Tetrads were dissected for sporulated diploids (Y2454-E857K×BY4741 *orfΔ*) heterozygous for *spt16-E857K* and *jhd1Δ* or *rph1Δ*. In each case, double-mutant segregants are circled. (C) Rpd3S activity is detrimental in *spt16-E857K bur2Δ* mutant cells. (top) Diploid cells heterozygous for *spt16-E857K, bur2Δ*, and *rco1Δ* were sporulated and resulting tetrads were dissected. *spt16-E857K bur2Δ rco1Δ* triple-mutant segregants are circled, while the positions of *spt16-E857K bur2Δ* double-mutant segregants are marked with squares. (bottom) Representative *bur2Δ* and *bur2Δ rco1Δ* segregants were grown to stationary phase in liquid culture and normalized for cell concentration, and 10-fold serial dilutions were spotted on rich solid medium for growth at the indicated temperatures. (D) Rpd3S activity inhibits *spt16-E857K* mutant cells lacking normal Paf1C activity. (top) Diploid calls heterozygous for *spt16-E857K*, *ctr9Δ*, and *rco1Δ* were sporulated and resulting tetrads were dissected. *spt16-E857K ctr9Δ rco1Δ* triple-mutant segregants are circled, while the positions of *spt16-E857K ctr9Δ* double-mutant segregants are marked with squares. (bottom) Representative segregants with the indicated genotypes were grown to stationary phase in liquid culture and normalized for cell concentration, and 10-fold serial dilutions were spotted on rich solid medium for growth at the indicated temperatures.

To ensure that deletions of genes involved in Set2-mediated H3K36 methylation effects do not themselves have deleterious genetic interactions with *spt16-E857K*, we assessed double-mutant cells harboring *spt16-E857K* and either *set2Δ*, *sin3Δ*, *eaf3Δ*, or *rco1Δ*. In each case, the double mutant grew as well as each single mutant (data not shown). Standard genetic procedures were then used to determine whether Set2-mediated H3K36 methylation is inhibitory in cells mutant for both Bur kinase and Spt16. We found that, in contrast to the lethality of *spt16-E857K bur2Δ* double-mutant cells, *spt16-E857K bur2Δ set2Δ* triple-mutant segregants formed colonies, showing that Set2 is indeed inhibitory in *spt16-E857K* cells with impaired Bur-kinase activity (Figure 4A). Spt16 and Bur kinase therefore carry out a common function that is antagonized by the Set2 histone methyltransferase.

Yeast cells have two H3K36-specific demethylases that counteract Set2 methylation activity: Rph1, which demethylates trimethylated K36 and to a lesser extent dimethylated K36, and Jhd1, which demethylates di- and mono-methylated K36 [69], [70]. Increased expression of either Rph1 or Jhd1, and the resultant decreases in H3K36 methylation [69], [70], can bypass the essential requirement for Bur1 [69], observations consistent with the genetic findings reported here. We tested the converse situation, whether increased levels of H3K36 methylation due to *jhd1Δ* or *rph1Δ* deletion mutations [69], [70] would impair the growth of *spt16-E857K* mutant cells. However, double-mutant cells harbouring *spt16-E857K* and either *jhd1Δ* or *rph1Δ* grew as proficiently as the single-mutant cells (Figure 4B). Increased levels of H3K36 methylation are not inhibitory in *spt16-E857K* mutant cells.

The status of Rpd3, the deacetylase subunit of Rpd3S, has both positive and negative effects in cells harboring *spt16* mutant alleles different than those used here [8], [11]; interpretation is difficult, however, for Rpd3 is also part of another histone deacetylase complex [67], [71]. A subunit specific to Rpd3S is Rco1 [67], [71]. In cells lacking Rco1, the Eaf3 subunit fails to associate with the remaining Rpd3S subunits, thus preventing formation of functional Rpd3S [71]. An *rco1Δ* gene deletion alleviated the synthetic lethality between *spt16-E857K* and *bur2Δ* (Figure 4C), suggesting that histone deacetylation makes chromatin difficult for *spt16-E857K bur2Δ* double-mutant cells to transcribe.

Unlike the positive effects on *bur2Δ* cells seen for an *eaf3Δ* deletion [67], the *rco1Δ* deletion did not improve the slow growth of *bur2Δ* cells (Figure 4C); *spt16-E857K rco1Δ bur2Δ* triple-mutant cells also exhibited slow growth, much like that of a *bur2Δ* cells (Figure 4C). These observations suggest that the slow growth of *bur2Δ* cells results from a different impairment than that exposed by Rpd3S histone deacetylation.

### Rpd3S intensifies *spt16-E857K* genetic interactions with Paf1C mutations

The *bur2Δ* findings described above suggest that the Rpd3S histone deacetylase may also be inhibitory in *spt16-E857K* cells mutated for Bur-kinase effectors. We found that neither *spt16-E857K spt5-4 rco1Δ* nor *spt16-E857K spt5-194 rco1Δ* triple mutants grew better at high temperatures than their respective *spt16-E857K spt5* double mutants (Figure 3A). In contrast, *rco1Δ* did relieve the deleterious genetic interactions of *spt16-E857K* with Paf1C mutations (Figure 4D). This *rco1Δ* effect is incomplete, however, for the growth of the triple mutants remained impaired at temperatures permissive for the single mutants, suggesting that only a subset of Paf1C activities is opposed by Rpd3S-mediated histone deacetylation (data not shown). These genetic interactions suggest that Rpd3S histone deacetylation makes Spt16 activity important, and imply that the Spt16-E857K mutant protein operates inefficiently with deacetylated histones.

### Rpd3S intensifies *spt16-E857K* genetic interactions with the histone chaperone HirC

**Figure 5:**
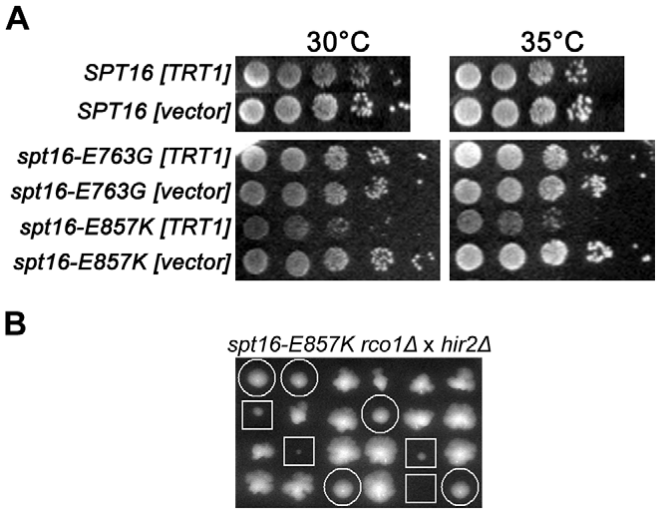
Rpd3S is detrimental in *spt16-E857K* cells with altered histone expression. (A) Increased expression of histones H2A–H2B impairs *spt16-E857K* cells. Cells of strains Y2454 and Y2454-E857K harboring the high-copy plasmid YEp24-TRT1, which carries the *HTA1–HTB1* histone-gene pair, were grown to stationary phase in liquid culture and normalized for cell concentration, and 10-fold serial dilutions were spotted on solid selective medium for growth at the indicated temperatures. (B) Rpd3S activity inhibits *spt16-E857K* cells lacking the histone-gene co-repressor HirC. Diploid cells (JS338×BY4741 *hir2Δ*) heterozygous for *spt16-E857K*, *hir2Δ*, and *rco1Δ* were sporulated, and the resulting tetrads were dissected (spores arranged vertically). The *spt16-E857K hir2Δ rco1Δ* triple mutant segregants (white circles) grew significantly better than the *spt16-E857K hir2Δ RCO1* double-mutant segregants (white squares).

The *bur2*, *spt4*, and *cdc73* (Paf1C) deletion mutations shown here to have deleterious genetic interactions with *spt16-E857K* are also deleterious in combination with mutations inactivating HirC, a histone chaperone [11], [72]. The HirC subunits Hir1 and Hir2 are structural orthologs of portions of the metazoan HIRA protein and, like HIRA, facilitate replication-independent histone deposition onto DNA [73], [74]. HirC is usually dispensible, but becomes very important in cells harbouring certain temperature-sensitive *spt16* mutant alleles [11]. Likewise, using the plasmid-loss procedure described above we found that HirC activity is critical for cells relying on the Spt16-E857K protein. In one derivative from our screen the deleterious genetic effects with *spt16-E857K* were due to the substitution mutation *hir2-L747R*; this L747R substitution affects the Hir2 HIRA domain, conserved among HIRA orthologs and thought to be important for protein function. In another derivative the genetic interaction was due to the mutation *hir1-L760P*; this L760P substitution affects the part of Hir1 involved in Hir2 binding [75], and may therefore weaken the physical interaction between Hir1 and Hir2. Consistent with this interpretation, a low-copy *HIR2* plasmid alleviated the synthetic lethality caused by *hir1-L760P* (data not shown). Our screen also identified a mutation affecting the HirC subunit Hir3 (data not shown). HirC mutations cause increased histone gene expression [76], and we found that increased expression of histones H2A–H2B from a high-copy plasmid [77] impairs *spt16-E857K* cells with normal HirC activity (Figure 5A), suggesting that the deleterious genetic effects of HirC mutations seen here are at least partially due to altered histone abundance and/or stoichiometry. Such histone alterations caused by HirC mutations may well affect transcription-related nucleosome disassembly, because the growth impairment of *spt16-E857K hir2Δ* cells was relieved by an *rco1Δ* mutation eliminating Rpd3S activity (Figure 5B). A role for HirC in transcription-linked nucleosome disassembly is also indicated by other findings [78]. These observations are consistent with the idea that, in addition to Spt16 (FACT) and Paf1C, histone expression exerts a third influence on transcription-linked nucleosome disassembly.

### The histone acetyltransferases Gcn5 and Esa1 facilitate Spt16-E857K activity

The above results suggest that Spt16 helps to overcome the repressive effects of deacetylated histones that result from Rpd3S activity. Spt16 may therefore interact with histone acetyltransferases (HATs) that acetylate the histones deacetylated by Rpd3S during the previous round of transcription. The HATs SAGA and NuA4 are known to mediate transcription-linked histone acetylation [79], [80]. We therefore assessed the growth of *spt16-E857K* cells harbouring mutations affecting these two HATs and other HATs implicated in transcription.

Sas3, Elp3 and Gcn5 are the catalytic subunits of HAT complexes, and deletion of their genes eliminates the HAT activity of NuA3 (*sas3Δ*) [81], Elongator (*elp3Δ*) [82] and SAGA, SLIK/SALSA, and ADA (*gcn5Δ*) [83]. Esa1 is the catalytic subunit of the HAT NuA4; the *ESA1* gene is essential, so we used the substitution mutation *esa1-L254P*, which enfeebles Esa1 [84], [85]. We saw no significant growth impairment for *spt16-E857K* in combination with *sas3Δ*, *elp3Δ*, *gcn5Δ*, or *esa1-L254P* (data not shown). This is another indication that *spt16-E857K* exerts more selective effects on Spt16 activity than does a temperature-sensitive mutation such as *spt16-11*, which has deleterious genetic interactions with the same HAT mutations [11].

**Figure 6:**
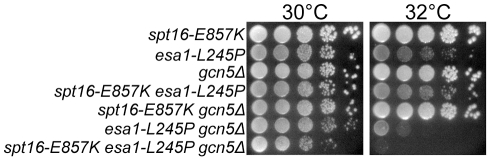
The Esa1 and Gcn5 histone acetyltransferases have an overlapping activity that benefits *spt16-E857K* mutant cells. Haploid *spt16 esa1-L254P* derivatives, created by several backcrosses of *esa1-L254P* (from strain LPY3500) into the Y2454 genetic background, were crossed with *gcn5Δ* mutant cells. Representative segregants with the indicated genotypes were grown to stationary phase and normalized for cell concentration, and 10-fold serial dilutions were spotted on rich solid medium for further growth at the indicated temperatures.

To test the possibility of overlapping HAT functions, we evaluated triple-mutant cells containing *spt16-E857K*, *esa1-L254P*, and either *gcn5Δ* or *sas3Δ* (the combination of *gcn5Δ* and *sas3Δ* is itself lethal) [86]. The *spt16-E857K esa1-L254P sas3Δ* triple mutants grew as well as the single or double mutants, indicating a lack of genetic interaction among these mutations (data not shown). In marked contrast, *spt16-E857K esa1-L254P gcn5Δ* triple-mutant cells grew very poorly, indicating a common functional role for the affected proteins (Figure 6). A milder genetic interaction was seen between *esa1-L254P* and *gcn5Δ* in cells with normal Spt16 function, as previously reported [80]. In each case, growth was more impaired at higher temperatures. The growth impairment did not seem to be a consequence of activation of an S-phase or M-phase checkpoint, for the *spt16-E857K esa1-L254P gcn5Δ* triple-mutant populations failed to accumulate cells with the large-budded morphology typical of cells with these checkpoints activated (data not shown). It is therefore likely that the growth defects of *esa1-L254P gcn5Δ* double-mutant and *spt16-E857K esa1-L254P gcn5Δ* triple-mutant cells result from impaired transcription.

### Spt16-E857K associates normally with RNA polymerase II, but poorly with histones

**Figure 7:**
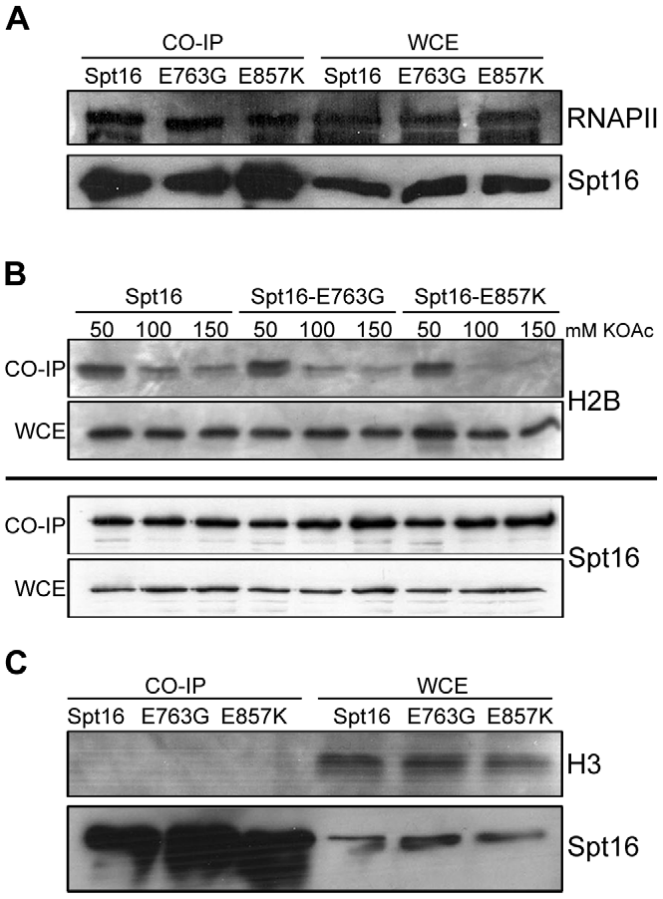
Spt16-E857K mutant protein associates normally with RNAPII, but poorly with histones. Extracts of cells (strains JS118, JS119, and JS120) with functional C-terminally S-tagged versions of Spt16 were treated with S-protein agarose beads, and the bound material was resolved electrophoretically, blotted, and probed with S-protein-HRP and with specific antibodies. (A) Spt16 proteins pull down RNAPII. S-tagged Spt16 and co-purifying material was resolved, blotted, and probed with antibodies against the RNAPII carboxy-terminal domain (CTD). (B) Spt16 association with histone H2B is weakened by the *spt16-E857K* mutation. S-tagged Spt16 and material co-purifying from extracts made at different potassium acetate concentrations was resolved, blotted, and probed with antibodies against H2B. (C) Spt16 proteins do not pull down histone H3. Material was assessed as in (B) except that antibodies against H3 were used.

Spt16 exists in complexes with RNAPII and many other proteins involved in transcription elongation [15], [64]. We found that RNAPII co-purified equally well with Spt16-E857K, Spt16-E763G, and normal Spt16 proteins (Figure 7A), indicating that these mutant Spt16 proteins retain normal RNAPII interactions.

Spt16 can bind H2A–H2B dimers [5]. We therefore assessed the interaction of histone H2B with Spt16-E857K, and Spt16-E763G, by co-purification (pull-down) assays. Interaction between Spt16 and histones is likely to be transient, so pull-downs were carried out over a range of potassium acetate concentrations to maximize the detection of weak interactions and indicate interaction strength. H2B co-purified with normal Spt16, Spt16-E763G, and Spt16-E857K, with substantially more H2B recovered in the 50 mM potassium acetate pull-downs than in the 100 mM or 150 mM pull-downs (Figure 7B). At higher potassium acetate concentrations less H2B co-purified with the Spt16-E857K mutant protein than with either Spt16 or Spt16-E763G, indicating that the interaction between Spt16-E857K and histone H2B is weak compared to that of normal Spt16 and Spt16-E763G.

Co-purification of histone H2B with Spt16 could be the result of interactions with H2A–H2B dimers and/or with intact nucleosomes. However, no histone H3 was detected in any of the Spt16 pull-downs (Figure 7C). Thus our co-purifications probably detected Spt16 interaction with free H2A–H2B dimers rather than with intact nucleosomes. This interpretation is consistent with the proposed role for Spt16 as a histone chaperone that extracts one H2A–H2B dimer from a nucleosome to allow passage of the transcription elongation complex [4]. As a result of its weaker histone-binding activity, however, Spt16-E857K is unable to facilitate effective nucleosome reassembly.

## Discussion

The histone chaperone FACT is involved in the elongation stages of RNAPII-mediated gene transcription, facilitating the dismantling and reassembly of nucleosomes encountered by the transcription complex [4]. For the Spt16 component of yeast FACT, several substitution mutations have been identified that impair transcription-linked nucleosome reassembly but preserve the essential functions of Spt16, and are thus selective in their effects (Table 1). We report here that one of these substitution mutations, *spt16-E857K*, also compromises an important histone-related activity for transcription elongation, as indicated by biochemical and genetic interactions. Co-purification studies showed that the Spt16-E857K mutant protein, while unaffected for interaction with RNAPII, is impaired for interaction with histone H2B, which probably reflects decreased interaction with the H2A–H2B heterodimer. The *spt16-E857K* mutation has deleterious genetic interactions with mutations affecting the transcription elongation factors Bur kinase, Spt4–Spt5, and Paf1C. Most of these mutations, like *spt16-E857K* itself, also impair transcription-linked nucleosome reassembly as indicated by cryptic-promoter activity [25], [60], [64]. In light of the evidence for functionally distinct classes of cryptic promoters [25], the poor cell growth of *spt16-E57K* cells harboring other nucleosome-reassembly mutations could in principle reflect an additive effect resulting in growth inhibition due to progressively more impaired nucleosome reassembly. However, our finding that an Rpd3S histone-deacetylase mutation, which also activates cryptic promoters [25], [71], alleviates the growth defect of *spt16-E857K* cells that are also mutant for Bur kinase or Paf1C is more consistent with models that emphasizes the importance of histone acetylation for disassembly of a nucleosome encountered by the transcription elongation complex. Bur kinase, Spt4–Spt5, and Paf1C form a functional pathway: Bur phosphorylates Spt4–Spt5, which in turn recruits Paf1C to the transcription complex. Our findings indicate that this Bur/Spt4–Spt5/Paf1C pathway and FACT have overlapping nucleosome-related activities that are important for transcription elongation.

Bur kinase and Spt4–Spt5, which have been independently implicated genetically in facilitating the transcription of nucleosomal templates [58], [67], most likely exert this effect through Paf1C: yeast Paf1C helps to destabilize nucleosomes upon transcription induction, and human Paf1C, in cooperation with the histone acetyltransferase p300, facilitates *in vitro* transcription elongation on a chromatin template [63], [87]. The disassembly of normally hypoacetylated nucleosomes is presumably hindered by the combined effects of *spt16-E857K* plus a mutation affecting Paf1C or an upstream pathway component, resulting in impaired transcription elongation and consequent growth effects. Conversely, Rpd3S deacetylase inactivation, which increases the acetylated state of nucleosomes [67], [71], most likely allows more effective nucleosome disassembly even when Spt16 and Paf1C are functionally compromised. This interpretation, coupled with the findings that *spt16-E857K*, in Rpd3S-dependent fashion, and the Bur/Spt4–Spt5/Paf1C pathway have deleterious genetic interactions with the histone chaperone HirC (Figure 5B) [72], supports a model in which FACT, Paf1C, and HirC separately influence transcription-linked disassembly of Rpd3S-deacetylated nucleosomes.

Nucleosome acetylation status is important in *spt16-E857K* cells, as indicated not only by the effects of Rpd3S inactivation but also by the genetic interactions resulting from inactivation or mutation of the HAT enzymes Gcn5, the acetyltransferase subunit of SAGA, and Esa1, the acetyltransferase subunit of NuA4. Both SAGA and NuA4 supply acetyltransferase activity during transcription elongation [79], [80], and both can facilitate the transcription of a nucleosomal template *in vitro* [88]. Thus acetylation is important for transcription-linked nucleosome disassembly, and becomes even more important in *spt16-E857K* cells. The genetic interactions between *spt16-E857K* and Bur/Spt4–Spt5/Paf1C pathway mutations suggest that FACT and/or these elongation factors may mediate transcription-linked nucleosome acetylation by the SAGA and/or NuA4 HAT complexes.

A Bur/Spt4–Spt5/Paf1C activity that is unlikely to be involved in the genetic interactions reported here is the stimulation of Set2 methyltransferase to convert H3K36me2 to H3K36me3. Nucleosomal H3K36me2 recruits Rpd3S *in vitro* and *in vivo* for the restoration of chromatin repression; H3K36me3 also binds Rpd3S *in vitro*, but its role *in vivo* is unclear [68], [89]. H3K36me3 abundance is significantly decreased by several mutations affecting Bur kinase and Paf1C [66], [90]. In contrast, the *spt4Δ*, *spt5-4*, and *spt5-194* mutations that also have deleterious genetic interactions with *spt16-E857K* leave H3K36me3 levels unaffected [25], [66]. Therefore, H3K36me3 status is not correlated with deleterious genetic interactions with *spt16-E857K*. Similar considerations dispense with other transcription-related activities that depend on Paf1C, including histone methylation at H3K4 and H3K36, and maintenance of CTD Ser-2 phosphorylation levels [62]. We find that H3K4 methylation is normal in *spt16-E857K* mutant cells, the loss of H3K4 methylation or the Ser-2 kinase CTDK-1 fails to impair the growth of these *spt16* mutant cells, and the absence of H3K36 methylation is actually beneficial. Thus the roles of Paf1C in histone methylation and CTD Ser-2 phosphorylation are unlikely to be involved in the genetic interactions described here.

An Spt4–Spt5 activity that does have relevance to the genetic interactions described here is the regulation of Rpd3S localization. Genome-wide assessment shows that Rpd3S is not uniformly distributed along transcribed regions [91]. In contrast, in *spt4Δ* cells Rpd3S is more uniformly distributed, matching the distribution of RNAPII itself. This aberrant distribution may reflect the association of Rpd3S with the phosphorylated CTD of RNAPII, a recruitment step that precedes Rpd3S binding to methylated H3K36 in transcribed nucleosomes [92]. Without Spt4 to mediate transfer from the CTD to the transcribed nucleosome, Rpd3S may be ineffective at nucleosomal deacetylation. A similar situation may be brought about by the *spt5-4* and *spt5-194* point mutations used here. The lack of influence of Rpd3S on the deleterious genetic interactions between *spt16-E857K* and Spt4–Spt5 mutations, as found here, may be due to this aberrant Rpd3S management by the mutant elongation complex.

Cells relying on Spt16-E857K protein are impaired not only for transcription-linked nucleosome reassembly [42], but also for the disassembly of nucleosomes encountered by the transcription elongation complex, as evidenced by the genetic interactions described here with factors involved in transcription elongation. In contrast to the dominant negative effect of mutant FACT (containing the Spt16-E857K mutant protein) on nucleosome reassembly, however, the effects of Spt16-E857K on transcription-linked nucleosome disassembly are recessive, as demanded by the procedure used here to identify deleterious genetic interactions between *spt16-E857K* and mutations inactivating Bur2 and HirC. Therefore, mutant FACT competes poorly with normal FACT in the genetic assays of transcription-linked nucleosome disassembly, even though it competes effectively with normal FACT in the genetic assays of transcription-linked nucleosome reassembly. One model to account for this functional difference proposes that FACT association with the transcription elongation complex is contingent on nucleosome disassembly, and that the same FACT complex that associates with an elongation complex as a result of nucleosome disassembly is retained for reassembly of the same nucleosome. This model for FACT interactions extends earlier findings that histone retention during transcription elongation depends on Spt16 activity [7], [20].

The activities of Spt16 for nucleosome reassembly after passage of the transcription elongation complex and nucleosome disassembly as the transcription elongation complex encounters the next nucleosome may be genetically separable, as suggested by the genetic interactions of *spt16-E763G*, another substitution allele that impairs nucleosome reassembly. This *spt16* mutation did not affect histone H2B interactions, had no deleterious genetic interactions with the HirC mutations or histone H2A–H2B overexpression, caused only mild temperature sensitivity in combination with *bur2Δ*, and showed no interactions with any of the other *bur1* mutant alleles (Figures 1C and 7B, and data not shown). This mutant allele also has a minimal extended phenotype (Table 1). These genetic distinctions suggest that Spt16-mediated transcription-linked nucleosome disassembly is not simply transcription-linked nucleosome reassembly run in reverse.

More striking *spt16* allele differences involve *spt16-E857Q*, a substitution mutant allele that relieves the cold sensitivity of a histone H3 mutation [13]. This mutation, causing a glutamine substitution at residue 857, fails to activate cryptic promoters and lacks genetic interactions with *bur2Δ*, while *spt16-E857K*, encoding a lysine substitution at residue 857, fails to alleviate the cold sensitivity of the H3 mutation (unpublished observations) [14]. These findings, plus others [14], show that the segment of the Spt16 protein encompassing residues 857 and 763 has multiple nucleosome-related activities.

Although almost the entire *SPT16* ORF was subjected to mutagenesis in the identification of the *spt16* mutant alleles studied here and listed in Table 1, the mutations found to have dominant effects on transcription-linked nucleosome reassembly, including *spt16-E857K* and *spt16-E763G*, alter only a limited segment approximating the middle domain of the large Spt16 polypeptide [4]. A bioinformatics approach [93] that predicts whether a polypeptide sequence has a folded structure resembling any of the known folds in the PDB protein-structure database indicates, with very high confidence, that the segment of Spt16 encompassing the sequence changes in Table 1 adopts the configuration of a double PH domain. This structural motif comprises two Pleckstrin Homology (PH) domains, each a 7-strand anti-parallel β-barrel structure capped at one end by a helix, oriented similarly and intimately associated with each other. The double PH domain predicted for Spt16 strongly resembles that determined experimentally for Pob3, the binding partner of Spt16 in yeast FACT [94]. Thus both components of FACT may contain a double PH domain. Another protein known to have a double PH domain is Rtt106, a histone chaperone (like Spt16 and Pob3) that is involved in transcription [95], [96]. Deletion of the gene encoding Rtt106 is deleterious for cells relying on the Spt16-E857K mutant protein (unpublished observation), suggesting that these structurally related proteins have related functions. The segment of Spt16 housing the predicted double PH domain thus has several transcription-related activities.

## Materials and Methods

### Strains

Strains used in this work are listed in Table S1; all are derivatives of S288C. Most of the deletion strains used were from the deletion collection [97]; Paf1C deletions [85], [90], [98] were a gift K. Arndt (Univ. of Pittsburgh), and the *set1*Δ deletion strain [80] was from A. Hinnebusch (NIH). The *spt4Δ* strain JS34 was obtained from a cross of strain Y2454 [99] with the BY4741-derived *spt4Δ::kanMX4* deletion-collection strain, which eliminated an extra, uncharacterized *kanMX4* insertion. All deletion strains used for direct analysis were confirmed using PCR. Strain AFO400 was derived by three backcrosses of strain KanB316-A4HA and derivatives to strain GHY1088, followed by transformation of an appropriate haploid segregant with pRS314-spt16-E857K. To create strain bur1Δ68Δ3C, one copy of *BUR1* was replaced with a *natMX4* cassette in strain KanBd and this derivative was transformed with pRS316-A4 and pGP161 and sporulated. Strains JS312 through JS329 are plasmid-shuffle derivatives in which pGP161 was replaced by versions of *BUR1* on pRS315-based plasmids, after which pRS316-A4 was replaced by versions of *SPT16* on pRS314-based plasmids. Before use, *esa1-L254P* segregants derived from strain LPY3500 were backcrossed three times into the Y2454 genetic background. Strains JS337 and JS338 have *rco1Δ::kanMX4* replaced by *rco1Δ::Ura3MX4* using the marker-swap plasmid pAG60 [100]. For analysis of *rad6Δ* effects, the *rad6Δ::kanMX4* gene-replacement allele from the BY4741-derived deletion strain was amplified by PCR and transformed into Y2454-E857K×BY4741 diploid cells. The resulting *spt16-E857K rad6Δ* heterozygous diploid cells, confirmed using PCR, were put through meiosis and sporulation, and fresh *rad6Δ* single-mutant and *spt16-E857K rad6Δ* double-mutant segregants were identified at 30°C and analyzed. Standard procedures were used for cell transformations, PCR, tetrad analysis, plasmid loss, and plasmid preparation.

### Plasmids

The plasmids used in this study are listed in Table S2. pRS314- and pRS315-based versions of *SPT16*, *spt16-E857K*, and *spt16-E763G* were created by subcloning from the pRS316-based versions [42]. S-tagged versions of each of the pRS315-based plasmids were created by insertion of PCR-amplified DNA containing S- and His_6_ tags from pET32, obtained using primers with XbaI adaptors, into an engineered XbaI site at the 3′ end of the *SPT16* ORF. The low-copy *LEU2* genomic library p366 was a gift from B. Andrews (University of Toronto). The low-copy *BUR1 TRP1* plasmid pGP161 was a gift from G. Prelich (Albert Einstein College of Medicine). pRS315-based *BUR1* and *bur1* mutant plasmids [49] were gifts from S. Buratowski (Harvard University). Plasmid pYL102 (HA-tagged H2B) [101] was a gift from R. Kornberg (Stanford University). Plasmid pAG60 was obtained from EUROSCARF.

### Mutant selection and identification

Plasmid-borne mutant alleles of the *SPT16* gene that, in dominant fashion, bring about transcription from otherwise repressed ‘cryptic’ promoters were created and selected as described [42]. DNA sequence obtained for the mutagenized region of such plasmids, from several independent mutagenesis runs, often identified a mutation encoding the substitution of glutamate at position 763 with glycine, which generates the sought-after phenotype [42]. Mutations causing this E763G substitution abolish a *Hin*dIII restriction site, allowing additional gap-repaired plasmids with the desired dominant effect to be screened by restriction analysis. Plasmids lacking a codon-763 mutation harbored a range of dominant mutant alleles, and fragment-swap plasmid reconstructions were used to eliminate substitutions with neutral effects. Phenotypes of mutant alleles, on low-copy pRS316-based plasmids, were assessed in FY2393 (*SPT16*) and KanB *(spt16Δ*) cells. Despite the dominant effect on reporter-gene expression used for selection, lower-case nomenclature is used here for these novel *spt16* mutant alleles because several phenotypes described below and previously [42] are recessive.

### Synthetic-lethal and library screening

Cells of strain AFO400, chromosomally deleted for *SPT16* but with viability maintained by separate *SPT16* and *spt16-E857K* plasmids, were subjected to UV mutagenesis, grown at 30°C, and screened for red/white colony sectoring as described [46], [47]. Approximately 70,000 colonies were screened at 30°C for derivatives that failed to grow after loss of the *SPT16 ADE3 URA3 GAL1pr-CEN4* plasmid pSLCDC68, and thus failed to produce red/white sectored colonies; galactose growth medium was used to destabilize pSLCDC68 inheritance [102]. A plasmid-shuffle procedure ruled out instances in which additional mutations in the *spt16-E857K* gene were responsible for the reliance on pSLCDC68. To identify the chromosomal gene responsible for the synthetic lethality with *spt16-E857K*, derivatives were transformed with the *LEU2*-based centromeric yeast genomic library p366 and screened for complementation of the lethal phenotype as indicated by restoration of colony sectoring. Plasmid-borne genomic inserts were identified by insert-vector junction sequencing. Mutated genomic loci were retrieved by gap-repair [103] and sequenced.

### Protein extractions

Mid-log phase cells (2–5×10^6^ cells/ml) were harvested by centrifugation (2 min, 3000 rpm) and protein extracts were obtained by glass-bead lysis in disruption buffer [20 mM Tris-Cl pH 7.9, 10 mM MgCl_2_, 1 mM EDTA pH 8.0, 5% glycerol, 0.3 M (NH_4_)_2_SO_4_] containing PMSF (10 µl/ml) and protease inhibitors (Sigma-Aldrich; 100× protease inhibitor stock: 62.5 µg/ml antipain, 0.05 µg/ml chymostatin, 2.5 µg/ml leupeptin, and 5 µg/ml pepstatin). For pull-down experiments, cells were washed once with ddH_2_O and resuspended in 500 µl of Co-IP Buffer (50 mM Tris-Cl pH 7.4, 50 to 150 mM KOAc, 5 mM EDTA pH 8.0, 0.1% Triton X-100, 10% glycerol, 1 mM NaN_3_) containing protease inhibitors and PMSF, and protein extracts were obtained by glass-bead lysis. Protein concentrations were quantified using the Bradford assay (BioRad) with a bovine serum albumin (BSA) standard.

### Co-purifications

Interactions between Spt16 and Nhp6, a transiently associating FACT subunit, can be detected in buffers with potassium acetate, but not with sodium chloride [104]. Therefore, buffers for pull-down studies contained potassium acetate and had low ionic strength. For Spt16 pull-downs, 500 µg of protein extract in Co-IP buffer with the desired salt concentration was added to 200 µl of a 50% slurry of S-protein agarose beads (Novagen, Madison, WI) in Co-IP buffer and incubated for 2 h with rotation. S-tagged Spt16 and associated proteins bound to the beads were washed 3 times with 1 ml Co-IP buffer and eluted in 50 µl of 2× Laemmli buffer by incubation for 10 min at 100°C.

### Western blotting

Proteins were resolved by SDS-PAGE and transferred to PVDF membrane (BioRad). Membranes with associated proteins were blocked overnight at room temperature in 10% milk (Carnation non-fat milk powder) or 5% BSA (when using antibodies against the RNAPII CTD) in 0.1% TBS-T buffer (10 mM Tris-HCl pH 7.4, 140 mM NaCl, 0.1% Tween 20), and washed 3×10 min in 0.1% TBS-T. Incubation with primary antibodies (in 0.1% TBS-T) was then carried out as follows: anti-HA (12CA5; Abcam, Cambridge, MA, or Santa Cruz Biotechnologies, Santa Cruz, CA) 1∶2000 in 1% milk, 2 h; anti-H3 (ab1791, Abcam) 1∶5000 in 1% milk, 1.5 h; anti-H3K4triMe (ab8580, Abcam) 1∶2000 in 1% milk, 2 h; anti-RNAPII (4H8, ab5408, Abcam) 1∶15,000 in 5% BSA, 2 h; and S-protein-HRP (Novagen) 1∶2000 in 1% milk, 2 h. Membranes were then washed 3×10 min in 0.1% TBS-T, followed by incubation with secondary antibodies (in 1% milk, 0.1% TBS-T for 2 h): goat anti-rabbit-HRP (Kirkegaard and Perry Laboratories, Gaithersburg, MD) at 1∶5000 (anti-H3 and anti-H3K4me3 blots), or goat anti-mouse-HRP (Santa Cruz) at 1∶2000 (anti-HA and anti-RNAPII blots). Membranes were then washed 3×10 min in 0.1% TBS-T, and bound antibody was detected using either the LumiGlo Chemiluminescent (Kirkegaard and Perry) or Western Lightning Chemiluminescent Reagent Plus (Perkin Elmer, Waltham, MA) substrate system.

## Supporting Information

Table S1 Yeast strains.
(DOC)

Table S2 Plasmids.
(DOC)
