# Supplementary material for: FACT, the Bur Kinase Pathway, and the Histone Co-Repressor HirC Have Overlapping Nucleosome-Related Roles in Yeast Transcription Elongation
Source: PLoS One. 2011 Oct 12;6(10):e25644. doi: 10.1371/journal.pone.0025644 (PMC3192111; doi:10.1371/journal.pone.0025644)
Supplement: Table S1 — Yeast strains. (DOC) [file pone.0025644.s001.doc]

**Table S1. Yeast strains**

| **Strain** | **Genotype** | **Source** |
| --- | --- | --- |
| orfΔ (1) | *MAT***a** *met15Δ0 his3Δ1 leu2Δ0 ura3Δ0 xyzΔ::kanMX4* | BY4741 deletions [97] |
| FY362 | *MAT***a** *his4-912δ ade8-104 spt5-194* | F. Winston, Harvard Univ. |
| FY1668 | *MAT***a** *his4-912δ lys2-128δ spt5-4* | [60] |
| GHY1088 | *MATα ade2Δ::hisG ade3Δ::hisG his4-912δ lys2-128δ leu2-Δ1 ura3-52* | G. Hartzog, Univ. Cal. Santa Cruz |
| DG183 | *set1∆::hphMX4* BY4741 derivative | [80] |
| KY688 | *MAT*a *lys2Δ0 ura3Δ0 trp1Δ63 cdc73Δ::kanMX4* | K. Arndt, Univ. Pittsburgh |
| KY690 | *MAT***a** *lys2-128δ leu2Δ1 ura3-52 leo1Δ::URA3* | [64] |
| KY693 | *MAT***a** *leu2Δ1 ura3-52 trp1Δ63 ctr9Δ::kanMX4* | [90] |
| KY802 | *MAT***a** *his3Δ200 lys2-173R2 ura3(-52 or Δ0) paf1Δ::URA3* | [98] |
| LPY3500 | *MAT***a** *his3Δ200 leu2-3,112 trp1Δ1 ura3-52 esa1Δ::HIS3 esa1-L254P:URA3* | [85] |
| AW11-9a | *MAT*α *his4-912δ lys2-128δ leu2-3,112 trp1-Δ1 ura3-52 suc2∆UAS* | [42] |
| FY2393 | *MAT***a** *lys2-128δ his3∆200 leu2∆1 trp1Δ63 ura3-52 kanMX-GAL1pr-FLO8-HIS3* | [42] |
| KanB-[plasmid] | *MAT***a** *his4-912δ lys2-128δ leu2-3,112 trp1-Δ1 ura3-52 ssd1-d cdc68Δ::kanMX4* [*spt16* or *SPT16* *CEN* plasmid] | [42] |
| AFO400 | *MATα ade2Δ ade3Δ his4-912δ lys2-128δ leu2-3,112 trp1-Δ1 ura3-52 spt16Δ::kanMX4* [pRS314-spt16-E857K] [pSLCDC68] | KanB316-A4HA × GHY1088 backcross segregant |
| AFO416 | *MATα ade2Δ ade3Δ his4-912δ lys2-128δ leu2-3,112 trp1-Δ1 ura3-52 spt16Δ::kanMX4* [pRS314-spt16-E857K] [pSLCDC68] *bur2-fs1* | AFO400 derivative |
| JS284 | *MATα ade2Δ ade3Δ his4-912δ lys2-128δ leu2-3,112 trp1-Δ1 ura3-52 spt16Δ::kanMX4* [pSLCDC68] | AFO400 plasmid-loss derivative |
| JS114 | *MATα ade2Δ ade3Δ his4-912δ lys2-128δ leu2-3,112 trp1-Δ1 ura3-52 spt16Δ::kanMX4* [pRS315-A4-S] | This study |
| JS115 | *MATα ade2Δ ade3Δ his4-912δ lys2-128δ leu2-3,112 trp1-Δ1 ura3-52 spt16Δ::kanMX4* [pRS315-E763G-S] | This study |
| JS116 | *MATα ade2Δ ade3Δ his4-912δ lys2-128δ leu2-3,112 trp1-Δ1 ura3-52 spt16Δ::kanMX4* [pRS315-E857K-S] | This study |
| JS118 | *MATα ade2Δ ade3Δ his4-912δ lys2-128δ leu2-3,112 trp1-Δ1 ura3-52 spt16Δ::kanMX4* [pRS315-A4-S] [pYL102] | JS114 transformant |
| JS119 | *MATα ade2Δ ade3Δ his4-912δ lys2-128δ leu2-3,112 trp1-Δ1 ura3-52 spt16Δ::kanMX4* [pRS315-E763G-S] [pYL102] | JS115 transformant |
| JS120 | *MATα ade2Δ ade3Δ his4-912δ lys2-128δ leu2-3,112 trp1-Δ1 ura3-52 spt16Δ::kanMX4* [pRS315-E857K-S] [pYL102] | JS116 transformant |
| bur1Δ68Δ3C | *MAT***a** *his4-912δ lys2-128δ leu2-3,112 trp1-Δ1 ura3-52 ssd1-d spt16Δ::kanMX4 bur1Δ::natMX4* [pRS316-A4] [pGP161] | This study |
| JS312 | *MAT***a** *his4-912δ lys2-128δ leu2-3,112 trp1-Δ1 ura3-52 ssd1-d spt16Δ::kanMX4 bur1Δ::natMX4* [pRS314-A4] [pRS315-BUR1-HA_3_] | This study |
| JS313 | *MAT***a** *his4-912δ lys2-128δ leu2-3,112 trp1-Δ1 ura3-52 ssd1-d spt16Δ::kanMX4 bur1Δ::natMX4* [pRS314-spt16-E763G] [pRS315-BUR1-HA_3_] | This study |
| JS314 | *MAT***a** *his4-912δ lys2-128δ leu2-3,112 trp1-Δ1 ura3-52 ssd1-d spt16Δ::kanMX4 bur1Δ::natMX4* [pRS314-spt16-E857K] [pRS315-BUR1-HA_3_] | This study |
| JS315 | *MAT***a** *his4-912δ lys2-128δ leu2-3,112 trp1-Δ1 ura3-52 ssd1-d spt16Δ::kanMX4 bur1Δ::natMX4* [pRS314-A4] [pRS315-bur1(1-393)-HA_3_] | This study |
| JS316 | *MAT***a** *his4-912δ lys2-128δ leu2-3,112 trp1-Δ1 ura3-52 ssd1-d spt16Δ::kanMX4 bur1Δ::natMX4* [pRS314-spt16-E763G] [pRS315-bur1(1-393)-HA_3_] | This study |
| JS317 | *MAT***a** *his4-912δ lys2-128δ leu2-3,112 trp1-Δ1 ura3-52 ssd1-d spt16Δ::kanMX4 bur1Δ::natMX4* [pRS314-spt16-E857K] [pRS315-bur1(1-393)-HA_3_] | This study |
| JS318 | *MAT***a** *his4-912δ lys2-128δ leu2-3,112 trp1-Δ1 ura3-52 ssd1-d spt16Δ::kanMX4 bur1Δ::natMX4* [pRS314-A4] [pRS315-bur1-T70A-HA_3_] | This study |
| JS319 | *MAT***a** *his4-912δ lys2-128δ leu2-3,112 trp1-Δ1 ura3-52 ssd1-d spt16Δ::kanMX4 bur1Δ::natMX4* [pRS314-spt16-E763G] [pRS315-bur1-T70A-HA_3_] | This study |
| JS320 | *MAT***a** *his4-912δ lys2-128δ leu2-3,112 trp1-Δ1 ura3-52 ssd1-d spt16Δ::kanMX4 bur1Δ::natMX4* [pRS314-spt16-E857K] [pRS315-bur1-T70A-HA_3_] | This study |
| JS321 | *MAT***a** *his4-912δ lys2-128δ leu2-3,112 trp1-Δ1 ura3-52 ssd1-d spt16Δ::kanMX4 bur1Δ::natMX4* [pRS314-A4] [pRS315-bur1-T240A-HA_3_] | This study |
| JS322 | *MAT***a** *his4-912δ lys2-128δ leu2-3,112 trp1-Δ1 ura3-52 ssd1-d spt16Δ::kanMX4 bur1Δ::natMX4* [pRS314-spt16-E763G] [pRS315-bur1-T240A-HA_3_] | This study |
| JS323 | *MAT***a** *his4-912δ lys2-128δ leu2-3,112 trp1-Δ1 ura3-52 ssd1-d spt16Δ::kanMX4 bur1Δ::natMX4* [pRS314-spt16-E857K] [pRS315-bur1-T240A-HA_3_] | This study |
| JS324 | *MAT***a** *his4-912δ lys2-128δ leu2-3,112 trp1-Δ1 ura3-52 ssd1-d spt16Δ::kanMX4 bur1Δ::natMX4* [pRS314-A4] [pRS315-bur1-23-HA_3_] | This study |
| JS325 | *MAT***a** *his4-912δ lys2-128δ leu2-3,112 trp1-Δ1 ura3-52 ssd1-d spt16Δ::kanMX4 bur1Δ::natMX4* [pRS314-spt16-E763G] [pRS315-bur1-23-HA_3_] | This study |
| JS326 | *MAT***a** *his4-912δ lys2-128δ leu2-3,112 trp1-Δ1 ura3-52 ssd1-d spt16Δ::kanMX4 bur1Δ::natMX4* [pRS314-spt16-E857K] [pRS315-bur1-23-HA_3_] | This study |
| JS327 | *MAT***a** *his4-912δ lys2-128δ leu2-3,112 trp1-Δ1 ura3-52 ssd1-d spt16Δ::kanMX4 bur1Δ::natMX4* [pRS314-A4] [pRS315-bur1-80-HA_3_] | This study |
| JS328 | *MAT***a** *his4-912δ lys2-128δ leu2-3,112 trp1-Δ1 ura3-52 ssd1-d spt16Δ::kanMX4 bur1Δ::natMX4* [pRS314-spt16-E763G] [pRS315-bur1-80-HA_3_] | This study |
| JS329 | *MAT***a** *his4-912δ lys2-128δ leu2-3,112 trp1-Δ1 ura3-52 ssd1-d spt16Δ::kanMX4 bur1Δ::natMX4* [pRS314-spt16-E857K] [pRS315-bur1-80-HA_3_] | This study |
| Y2454 | *MATα mfa1Δ::MFA1pr-HIS3 can1Δ his3Δ1 leu2Δ0 ura3Δ0 MET15 lys2Δ0* | [99] |
| Y2454-WT68 | *MATα mfa1Δ::MFA1pr-HIS3 can1Δ his3Δ1 leu2Δ0 ura3Δ0 MET15 lys2Δ0 SPT16:natMX4* | [42] |
| Y2454-E763G | *MATα mfa1Δ::MFA1pr-HIS3 can1Δ his3Δ1 leu2Δ0 ura3Δ0 MET15 lys2Δ0 spt16-E763G:natMX4* | [42] |
| Y2454-E857K | *MATα mfa1Δ::MFA1pr-HIS3 can1Δ his3Δ1 leu2Δ0 ura3Δ0 MET15 lys2Δ0 spt16-E857K:natMX4* | [42] |
| JS34 | *MAT***a** *MET15? his3Δ1 leu2Δ0 ura3Δ0 spt4Δ::kanMX4* | BY4741 spt4Δ × Y2454 segregant |
| JS330 | *MATα leu2Δ0 ura3Δ0 his3Δ1 lys2? met15? can1? (mfa1Δ::MFA1pr-HIS3)? spt16-E857K:natMX4 rco1Δ::kanMX4* | Y2454-E857K × rco1Δ segregant |
| JS338 | *MATα leu2Δ0 ura3Δ0 his3Δ1 lys2? met15? can1? (mfa1Δ::MFA1pr-HIS3)? spt16-E857K:natMX4 rco1Δ::URA3MX4* | JS330 derivative |

(1) Deletions used were *bur2Δ, ctk1Δ, set1∆, swd1Δ, swd3Δ, rad6Δ, spt4Δ, set2Δ, eaf3∆, rco1Δ, sin3Δ, rph1Δ, jhd1Δ, hir2∆, sas3Δ, elp3Δ, gcn5Δ*
