# Supplementary material for: FACT, the Bur Kinase Pathway, and the Histone Co-Repressor HirC Have Overlapping Nucleosome-Related Roles in Yeast Transcription Elongation
Source: PLoS One. 2011 Oct 12;6(10):e25644. doi: 10.1371/journal.pone.0025644 (PMC3192111; doi:10.1371/journal.pone.0025644)
Supplement: Table S2 — Plasmids. (DOC) [file pone.0025644.s002.doc]

Table S2. Plasmids

| **Plasmid name** | **Description** | **Source** |
| --- | --- | --- |
| pRS316-A4 | SPT16 URA3 CEN | [42] |
| pRS315-A4 | *SPT16 LEU2 CEN* | This study |
| pRS315-A4-S | C-terminally S-tagged *SPT16 LEU2 CEN* | This study |
| pRS314-A4 | *SPT16 TRP1 CEN* | This study |
| pRS315-E763G | *spt16-E763G LEU2 CEN* | This study |
| pRS315-E763G-S | C-terminally S-tagged *spt16-E763G LEU2 CEN* | This study |
| pRS314-E763G | *spt16-E763G TRP1 CEN* | This study |
| pRS315-E857K | *spt16-E857K LEU2 CEN* | This study |
| pRS315-E857K-S | C-terminally S-tagged *spt16-E857K LEU2 CEN* | This study |
| pRS314-E857K | *spt16-E857K TRP1 CEN* | This study |
| pSLCDC68 | *SPT16 ADE3 URA3 GAL1pr-CEN4* | pSLS1 [102] derivative; M. Dobson, Dalhousie Univ. |
| p366 | *LEU2 CEN* genomic library (9- to 12-kbp inserts) | B. Andrews, Univ. Toronto |
| pGP161 | *BUR1 TRP1 CEN* | G. Prelich, Albert Einstein Coll. Med. |
| pRS315-BUR1-HA_3_ | *BUR1 LEU2 CEN* | [49] |
| pRS315-bur1(1-393)-HA_3_ | *bur1(1-393) LEU2 CEN* | [49] |
| pRS315-bur1-T70A-HA_3_ | *bur1-T70A LEU2 CEN* | [49] |
| pRS315-bur1-T240A-HA_3_ | *bur1-T240A LEU2 CEN* | [49] |
| pRS315-bur1-80-HA_3_ | *bur1-80 LEU2 CEN* | [49] |
| pRS315-bur1-85-HA_3_ | *bur1-85 LEU2 CEN* | [49] |
| pRS315-bur1-35-HA_3_ | *bur1-35 LEU2 CEN* | [49] |
| pRS315-bur1-23-HA_3_ | bur1-23 *LEU2 CEN* | [49] |
| pRS315-bur1-51-HA_3_ | *bur1-51 LEU2 CEN* | [49] |
| pRS315-bur1-65-HA_3_ | *bur1-65 LEU2 CEN* | [49] |
| pRS315-bur1-78-HA_3_ | *bur1-75 LEU2 CEN* | [49] |
| YEp24-TRT1 | *HTA1–HTB1 URA3 2μ* | [77] |
| pYL102 | *prGAL10-HA-H2B TRP1 CEN* | [101] |
| pAG60 | *kanMX::Ura3MX4 Amp^R^* | [100] |
